# Development of an HPLC method with relative molar sensitivity based on ^1^H-qNMR to determine acteoside and pedaliin in dried sesame leaf powders and processed foods

**DOI:** 10.1371/journal.pone.0243175

**Published:** 2020-12-03

**Authors:** Takashi Ohtsuki, Kiyoaki Matsuoka, Yushiro Fuji, Yuzo Nishizaki, Naoko Masumoto, Naoki Sugimoto, Kyoko Sato, Hiroshi Matsufuji

**Affiliations:** 1 Department of Food Bioscience and Biotechnology, College of Bioresource Sciences, Nihon University, Fujisawa, Kanagawa, Japan; 2 Division of Food Additives, National Institute of Health Sciences, Kawasaki-ku, Kawasaki, Kanagawa, Japan; Higher Institute of Applied Sciences and Technology of Gabes University of Gabes, TUNISIA

## Abstract

A high-performance liquid chromatography (HPLC) method with relative molar sensitivity (RMS) based on ^1^H quantitative NMR spectroscopy (^1^H-qNMR) has been developed for food ingredients such as acteoside (verbascoside) and pedaliin (pedalitin-6-*O*-glucoside) without requiring authentic and identical standards as the reliable analytical methods. This method is used methyl 4-hydroxybenzoate (MHB) as an alternative reference standard. Each RMS is also calculated from the ratio of each analyte's molar absorption coefficient to that of MHB after correcting the purities of the analytes and reference standard by ^1^H-qNMR. Therefore, this method can quantify several analytes with metrological traceability to the International System of Units (SI) using the RMS and one alternative reference standard. In this study, the content of acteoside and pedaliin in several samples, such as dried sesame leaf powders and commercially processed foods, can be determined by the proposed RMS method and demonstrated in good agreement that obtained by a conventional method. Moreover, the proposed method yields analytical data with SI-traceability without the need for an authentic and identical analyte standard. Thus, the proposed RMS method is a useful and practical tool for determining acteoside and pedaliin in terms of the accuracy of quantitative values, the routine analysis, and the cost of reagents.

## Introduction

It is crucial to ensure the quality and safety of food sold domestically and internationally using analytical evaluations, and thus there is a need to develop highly reliable analytical methods for various food-related compounds. Quantification is commonly accomplished by purifying a sample and analyzing it with chromatographic methods such as high-performance liquid chromatography (HPLC). However, the sensitivity of HPLC methods depends on the molar absorption coefficient of the specific analyte. An authentic and identical analyte standard, such as certified reference material (CRM) with proven identity and purity determined by valid metrological procedures, is thus required to obtain reliable analytical results for each analyte. However, there are very few certified reference materials, and therefore quantification methods such as HPLC are typically used together with commercial standard products from reagent manufacturers as reference standards. Since the manufacturer guarantees these commercial standards' purity, it may not be metrologically accurate, resulting in questionable reliability of the obtained quantitative value. Additionally, for naturally derived compounds such as food-related compounds, not all reference standards used for quantification are commercially available, requiring the isolation and purification, or chemical synthesis, of these compounds used as reference standards for quantification. However, considerable effort is required to prepare purified or synthesized standards. Furthermore, even if a reference standard for quantifying a food compound is currently commercially _FTble, the reagent manufacturer may terminate the supply because it becomes economically unviable. Such restrictions and/or concerns regarding the availability of reference standards for analytes are significant obstacles for precise analyses and establishing analytical methods for food-related compounds.

Quantitative NMR using ^1^H NMR (^1^H-qNMR) is a powerful tool for quantifying analytes. Thus, this approach uses the primary ratio method and does not require standards identical to the analyte [1,2]. An analyte's content or concentration is determined using the ratio of the integral value of a specific signal of the analyte to that of an internal standard (IS). The analyte and IS's integral values directly proportional to the number of protons per resonance line times the molar concentration of the analyte and the IS, respectively. Therefore, the analyte's absolute quantitative value can be determined with metrological traceability to the International System of Units (SI) if a specific CRM is used as the IS in ^1^H-qNMR [3,4]. A HPLC method using the relative molar sensitivity (RMS) of ^1^H-qNMR (RMS method) was recently reported to quantify analytes using a reference standard different from the analyte [5]. RMS is calculated by the ratio of the molar absorption coefficient of the analyte to that of this alternative reference standard by correcting the analytes' purities using ^1^H-qNMR. Thus, this method allows the quantification of analytes with metrological traceability to the SI using RMS, a non-analyte reference standard, and the molecular weight ratio of the analyte to the alternative reference standard. This method has been applied to quantify food compounds [6], polycyclic aromatic hydrocarbons [7], and the main compounds of food additives [8–11] because it allows SI-traceability of the quantification value and utilizes the highly versatile separation performance of HPLC.

In previous studies, our group chemically characterized three iridoids and eight polyphenols in young sesame leaves (*Sesamum indicum* L.), including their biological activities such as 1,1-diphenyl-2-picrylhydrazyl radical scavenging, oxygen radical absorbance capacity, *in vitro* antiglycation activity, changes in the content of five compounds at each growth stage of sesame leaves, and the accumulation and subcellular localization of acteoside (verbascoside) in sesame plants [12–14]. Among the polyphenols identified, acteoside and pedaliin (pedalitin-6-*O*-glucoside) are respectively a major phenylethanol glycoside and a flavonoid glycoside in young sesame leaves. In particular, acteoside has been found to exhibit a high level of hepatoprotective activity [15], anti-inflammatory [16], antioxidant [17], protective properties against neurological diseases, such as Parkinson’s [18] and Alzheimer’s diseases [19,20], indicating that this compound is expected to be useful as a medicinal drug or seeds of medicine [13]. Generally, Chromatographic methods such as HPLC [21,22] and LC-MS/MS [23,24] were used to quantify acteoside in plant and non-plant biological samples. In this quantification, the reference standard for quantification of acteoside is commercially available, but it is relatively expensive. In addition, there is no CRM commercially available for acteoside worldwide. Moreover, although pedaliin has been previously reported in *Verbena Officinalis* [25] and *Dracocephalum tanguticum* [26] beside *Sesamum indicum*, neither an authentic reference standard for quantification nor a CRM or reagent, is commercially available for pedaliin. These limitations have hampered the establishment of reliable quantification methods for both compounds.

This study reports an HPLC method for acteoside and pedaliin with RMS based on ^1^H-qNMR that ensured the quantitative values' reliability. In addition, the method's performance using three dried sesame leaf powders, and two processed foods containing these compounds were evaluated.

## Materials and methods

### Samples and reagents

Two dried sesame leaf powders (dry powders 1 and 2) were prepared from the leaves of sesame plants (*Sesamum indicum* L.) of different heights cultivated in a greenhouse at the College of Bioresource Sciences, Nihon University (dry powder 1: 70 cm, dry powder 2: 40 cm). Another dried sesame leaf powder from *Sesamum indicum* L. (dry powder 3) was obtained from Wadaman Co. Ltd. (Kyoto, Japan). One processed food (green juice 1, product name: Gomawakaba aojiru), which contains powder of young sesame leaves, galactooligosaccharide, the extract of fermented germinated brown rice, cyclic oligosaccharide, was obtained from Wadaman Co. Ltd. A second processed food (green juice 2, product name: Aojiru douraku), which contains powder of young sesame leaves, digestion resistant dextrin, plant lactobacillus, was manufactured by Grand-sun Co., Ltd. (Tokyo, Japan). Methyl 4-hydroxybenzoate (MHB) was manufactured by Tokyo Chemical Industry Co., Ltd. (Tokyo, Japan). Acteoside reagent (analytical grade) was manufactured by Funakoshi Co., Ltd. (Tokyo, Japan). Pedaliin was isolated from young sesame leaf powder and was identified by spectral analyses and comparison with reported data. 2,2-dimethyl-2-silapentane-5-sulfonate-*d*6 sodium salt (DSS-*d*_6_) (purity: 92.4% ± 0.5%), the certified reference material, was obtained from Fujifilm Wako Pure Chemical Industries (Osaka, Japan) and was obtained from Fujifilm Wako Pure Chemical Industries (Osaka, Japan) and used as the internal standard for ^1^H-qNMR. Deuterated dimethyl sulfoxide (DMSO-*d*_6_) and deuterated methanol, manufactured by Kanto Kagaku Co., Inc. (Tokyo, Japan), were used as the ^1^H-qNMR solvent. Ultrapure water was purified to 18 MΩ cm using a Milli-Q (Merck, Tokyo, Japan). Other reagents and solvents were special grade or HPLC grade.

### Instruments

An AUW220D semi-micro balance (Shimadzu Corporation, Kyoto, Japan) was used to weigh the three dried sesame leaf powders and two processed foods, DSS-*d*_6_ stock solution, methanol-*d*_4_, and DMSO-*d*_6_. A BM-20 microbalance (A&D Company, Limited, Tokyo, Japan) was used to weigh acteoside reagent, isolated pedaliin, and MHB for ^1^H-qNMR analysis. Analytical HPLC was performed using a HPLC system comprising a SIL-20A autosampler, a LC-20AD liquid pump, a CTO-10AS column oven, an SPD-M20A multi-wavelength detector, and LabSolutions data-processing software (Shimadzu Corporation). Preparative HPLC was performed using an HPLC system comprising a LC-6AD liquid pump, a CTO-6A column oven, a SPD-10A_VP_ UV–visible absorbance detector (Shimadzu Corporation), and a Chromato-PRO integrator (Runtime Instruments, Tokyo, Japan). ^1^H-qNMR was performed using a JNM-ECA500 (proton resonance frequency: 500 MHz) instrument manufactured by JEOL (Tokyo, Japan).

### Isolation of pedaliin

Dried young sesame leaf powder (300 g) was added to 1.5 L of methanol and ultrasonicated. After centrifugation (5000 × *g*, 5 min), the supernatant was collected and concentrated using a rotary evaporator to obtain an extract (100 mL). The extracted was loaded on a Diaion HP-20 (Mitsubishi Chemical Co. Ltd., Tokyo, Japan) column (50 mm i.d. × 340 mm), then 2.5 L of methanol was passed through to obtain a methanol fraction (30.5 g). A 2.7 g aliquot was diluted with 50 mL of 70% methanol. After centrifugation (5000 × *g*, 5 min), the supernatant was allowed to stand for 12 hours at room temperature. The obtained precipitate was purified by preparative HPLC with monitoring at 280 nm using a Develosil ODS-UG-5 (4.6 mm i.d. × 250 mm, particle size 5 μm; Nomura Chemical, Tokyo, Japan) column at 45°C and a flow rate of 1.0 mL/min with 20% acetonitrile as the mobile phase to afford pedaliin (6.0 mg). Prepared pedaliin was completely characterized by spectral analysis and comparison with reported data [13].

### Determination of purity of acteoside, pedaliin, and MHB by ^1^H-qNMR

#### Preparation of standard solution for ^1^H-qNMR

The standard solution 1 for pedaliin and MHB in ^1^H-qNMR was prepared by precisely weighing 14.5 mg of DSS-*d*_6_ using a microelectronic balance and adding 66.8 g of DMSO-*d*_6_ as solvent (DSS-*d*_6_ concentration: 0.2006 mg/g). The standard solution 2 for acteoside in ^1^H-qNMR was prepared by precisely weighing 10.0 mg of DSS-*d*_6_ using a microelectronic balance and adding 46.1 g of methanol-*d*_4_ as solvent (DSS-*d*_6_ concentration: 0.2004 mg/g).

#### Preparation of test solution for ^1^H-qNMR

Each compound was precisely weighed and dissolved in 1 g of standard solutions 1 or 2 to prepare the test solutions: 15 mg of acteoside, 6 mg of pedaliin, and 14.1 mg of MHB. Approximately 0.6 mL of each solution was placed individually in an NMR sample tube with an outer diameter of 5 mm, and the tubes were sealed to prior to ^1^H-qNMR measurements.

#### ^1^H-qNMR measurement and calculation of the purity of each sample

^1^H-qNMR was performed using the following optimized parameters: irradiation frequency, 500 MHz; probe temperature, 25°C; spinning, off; number of scans, 8; spectral width, 15 ppm; auto filter, on (eight times); acquisition time, 4.37 s; relaxation delay, 60 s; pulse angle, 90°; free induction decay (FID) data points, 32 k; and ^13^C decoupling, multi-pulse decoupling with phase and frequency switching (MPF-8). The data were processed using the ^1^H-qNMR analysis software, Alice 2 for qNMR "PURITY" (JEOL RESONANCE Ltd., Tokyo, Japan). The signal integral value calculated with this software was used for quantitative analysis. For all chemical shifts, the proton signal of the DSS-*d*_6_ standard was set as the reference signal (0 ppm). The purity (%, P_sample_) of each sample was calculated using Eq (1):

$$P_{\mathrm{Sample}}=\frac{I_{\mathrm{Sample}}/H_{\mathrm{Sample}}}{I_{\mathrm{DSS}}/H_{\mathrm{DSS}}}\times \frac{M_{\mathrm{Sample}}/W_{\mathrm{Sample}}}{M_{\mathrm{DSS}}/C_{\mathrm{DSS}}}\times 100$$ (1)

where, I_Sample_ and I_DSS_ are the signal integral values for the sample and DSS-*d*_6_ (9.000), respectively. H_sample_ and H_DSS_ are the number of protons for the sample and DSS-*d*_6_ signals (CH_3_ × 3 = 9), respectively. M_Sample_ is molecular weight of the sample (i.e., 624.6 for acteoside, 478.4 for pedaliin, and 152.2 for MHB) and DSS-*d*_*6*_ (224.36), respectively. M_DSS_ is the molecular weight of DSS-*d*_6_ (224.36). W_Sample_ is the mass of the measurement sample (mg), and C_DSS_ is the DSS-*d*_6_ concentration of the ^1^H-qNMR standard solution.

### Calculation of the RMS of acteoside and pedaliin relative to MHB

Seven concentrations of acteoside, pedaliin, and MHB standard solutions (solvent: methanol) were prepared in the concentration range of 1.6–100 μmol/L based on purities calculated from ^1^H-qNMR data. These solutions (5 μL each) were analyzed by HPLC using an Xbridge C_18_ column (4.6 mm × 150 mm, particle size 5 μm, Waters Corporation, MA, USA). The mobile phase consisted of solvent A (0.1 vol% formic acid) and B (acetonitrile containing 0.1 vol% formic acid). The gradient conditions were: 5%–55% solvent B from 0–25 min, 55%–100% solvent B (25.01–35 min). After each measurement, the column was re-equilibrated for 5 min with 5% solvent B. The flow rate was kept at 1 mL/min. The column temperature was set to 40°C and the detector was operated at an absorbance wavelength of 340 nm for acteoside and pedaliin and at 254 nm for MHB.

The slope (molar absorption coefficient) of the calibration curve for each sample set passing through the origin was calculated, then the RMSs of acteoside and pedaliin relative to MHB were calculated from the ratio of the slopes of the calibration curves for acteoside or pedaliin to MHB, respectively.

### Quantification of acteoside and pedaliin by HPLC using each RMS

Each sample including three dried sesame leaf powders and two processed foods was precisely weighed and dissolved in 10 mL of 60 vol% methanol: 15 mg of dried sesame leaf powder 1, 11 mg of dried sesame leaf powders 2 and 3, and 11 mg of green juice powders 1 and 2. Following ultrasonication for 15 minutes and centrifugation (5000 x *g*) for 5 minutes, the supernatants were transferred to volumetric flasks. To each residue, 10 mL of 60 vol% methanol was added. The above-described procedure was repeated, and the supernatants were transferred to the respective volumetric flasks. Methanol was added to each volumetric flask to 20 mL to obtain the test stock solutions, which were appropriately diluted and filtered through 0.45 μm membrane filters to obtain the test solutions. These test solutions and the MHB standard solutions were analyzed using the HPLC conditions indicated, as mentioned above. The concentrations of acteoside and pedaliin in the test solutions were determined by integrating the peak areas of acteoside and pedaliin, and from the MHB calibration curve. Then, the contents of acteoside and pedaliin in each sample were calculated using Eq (2):

$$\mathrm{Content}\;(\mu g/\mathrm{kg})=\frac{C\times V\times M\times 10^{6}}{W\times 1000}\times \frac{1}{\mathrm{RMS}}$$ (2)

where C is the concentration of each analyte in the test solution obtained from the MHB calibration curve (μmol/L), V is the volume of test solution (mL), W is the sample weight (mg), and RMS is the RMS of acteoside or pedaliin to MHB.

### Quantification of acteoside and pedaliin by a conventional HPLC method

Using the test solutions prepared and the acteoside and pedaliin standard solutions prepared, the two analytes in each sample were quantified using the HPLC conditions as mentioned above. The concentrations of acteoside and pedaliin in the test solutions were determined by integrating the peak areas of acteoside and pedaliin and the calibration curve for each reference standard. Then, the concentrations of acteoside and pedaliin in each sample were calculated using Eq (3):

$$\mathrm{Content}\;(\mu g/\mathrm{kg})=\frac{C\times V\times 10^{6}}{W\times 1000}$$ (3)

## Results and discussion

### Purity determination of acteoside, pedaliin, and MHB by ^1^H-qNMR

**Fig 1:**
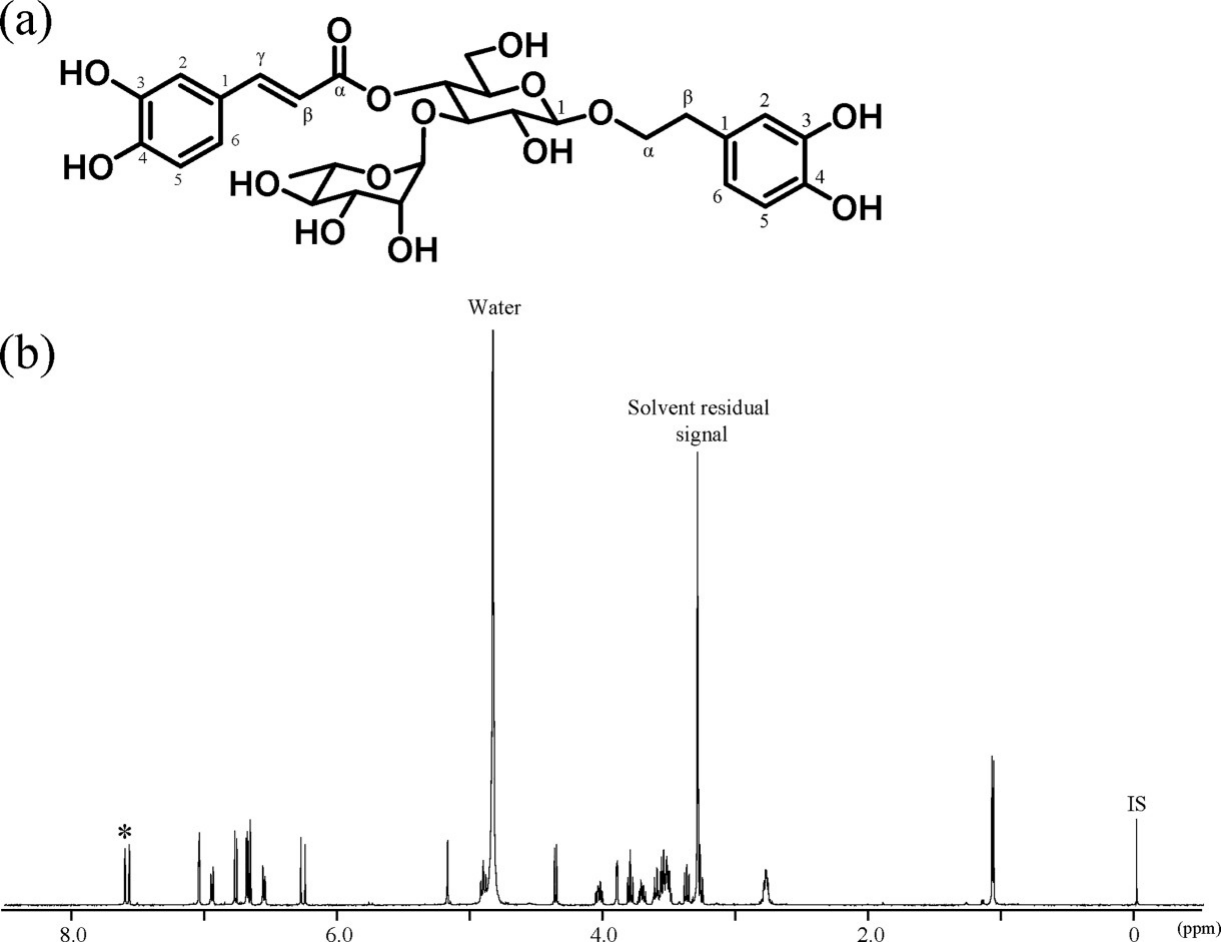
Chemical structure of acteoside, and its ^1^H-qNMR spectrum in methanol-*d*_4_ containing DSS-*d*_6_. IS, internal standard (DSS-*d*_6_).

In ^1^H-qNMR, the signal area intensity and the molar concentration of the reference compound and analyte are used to quantify the analyte concentration. Furthermore, using a reference compound with quantitative measurement traceability should improve the obtained quantitative values' reliability. To determine the purities of acteoside, pedaliin, and MHB, these compounds were first analyzed using ^1^H-qNMR measurements in triplicate. The chemical shifts (δ) are in ppm and referenced to the methyl proton signal of the DSS-*d*_6_ internal standard (δ 0 ppm). As shown in Fig 1, ^1^H NMR signals derived from acteoside were observed from δ 1.15 to 7.58 ppm.

Characteristic signals from the methyl groups at the 6-position of rhamnose (δ 1.15 ppm) and the β-position of the aglycon (δ 2.78 ppm), glucose and rhamnose (δ 3.00–5.20 ppm), the α′-position of the caffeoyl group (δ 6.25 ppm), the 6-position of the aglycone (δ 6.54 ppm), and the 2-position of the caffeoyl group (δ 7.03 ppm) were partially or fully overlapped by other intramolecular signals or signals from contaminants, making it difficult to set a suitable integration range for quantification. However, because the proton signal derived from the β′ position of the caffeoyl group (δ 7.58 ppm) was sufficiently separated from other signals in the molecule, this signal was applied for quantifying acteoside. As a result, the purity of acteoside calculated from this signal was 79.8%.

**Fig 2:**
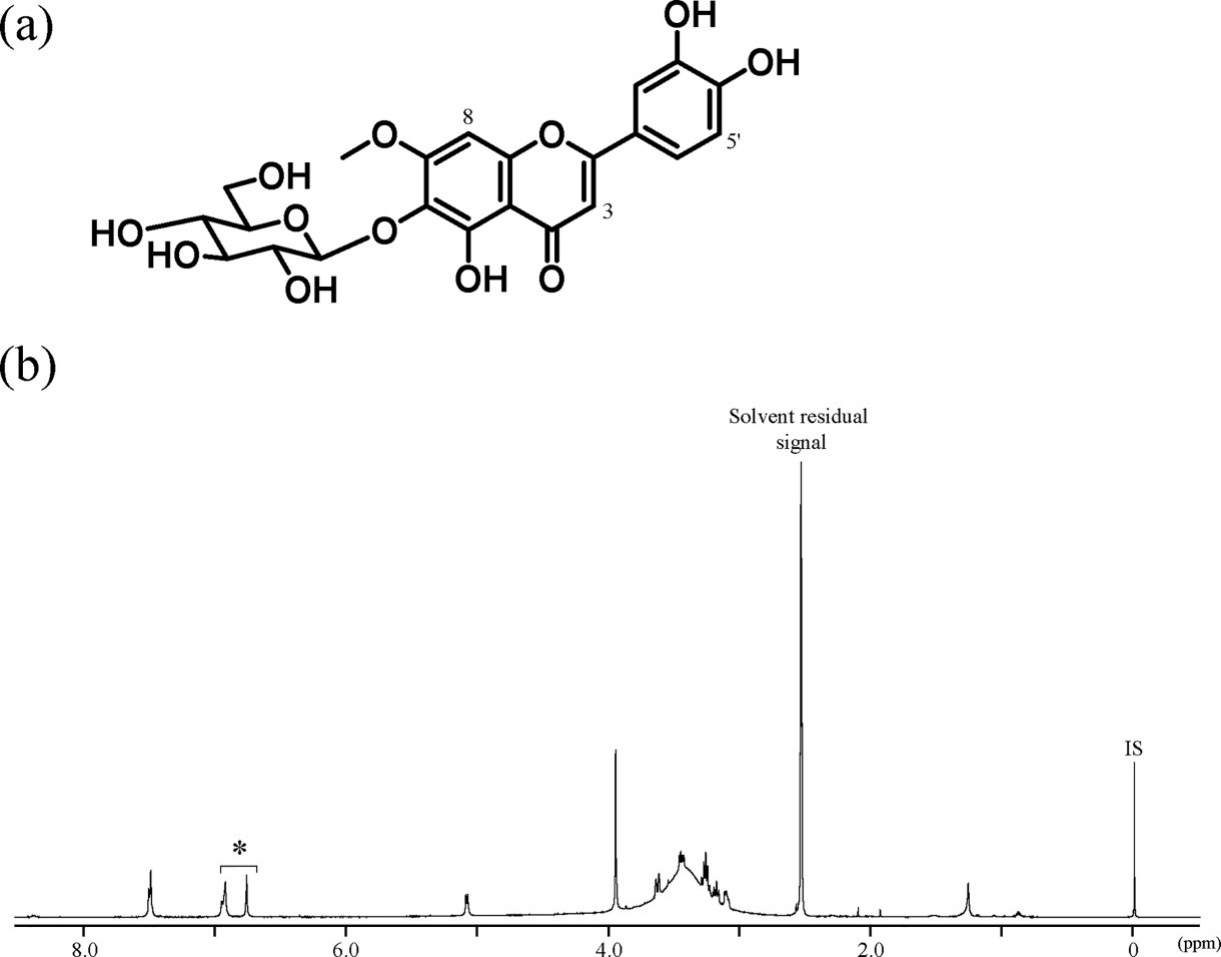
Chemical structure of pedaliin, and its ^1^H-qNMR spectrum in DMSO-*d*_6_ containing DSS-*d*_6_. IS, internal standard (DSS-*d*_6_).

For pedaliin, a series of signals were observed from δ 3.15 ppm to 7.41 ppm in the ^1^H-qNMR spectrum (Fig 2).

The proton resonances from glucose positions 2 to 6 (δ 3.15–3.62 ppm), the signal of the 7-position methoxy group of the aglycone (δ 3.87 ppm), and the 1-position of glucose (δ 5.07 ppm) were unsuitable for quantification due to the intensity of the signal derived from residual water. The proton signal (δ 7.41 ppm) from the 2′ and 6′ positions of the aglycone was also unsuitable for quantification because a signal derived from impurities was observed at the base of the peak. In contrast, although not fully separated, the resonances derived from the 3′(δ 6.74 ppm), 5′(δ 6.91 ppm), and 8′ (δ 6.90 ppm) positions of the aglycone could be integrated for quantification because there was no overlap with contaminant signals, allowing the purity of pedaliin to be calculated as 65.1%.

**Fig 3:**
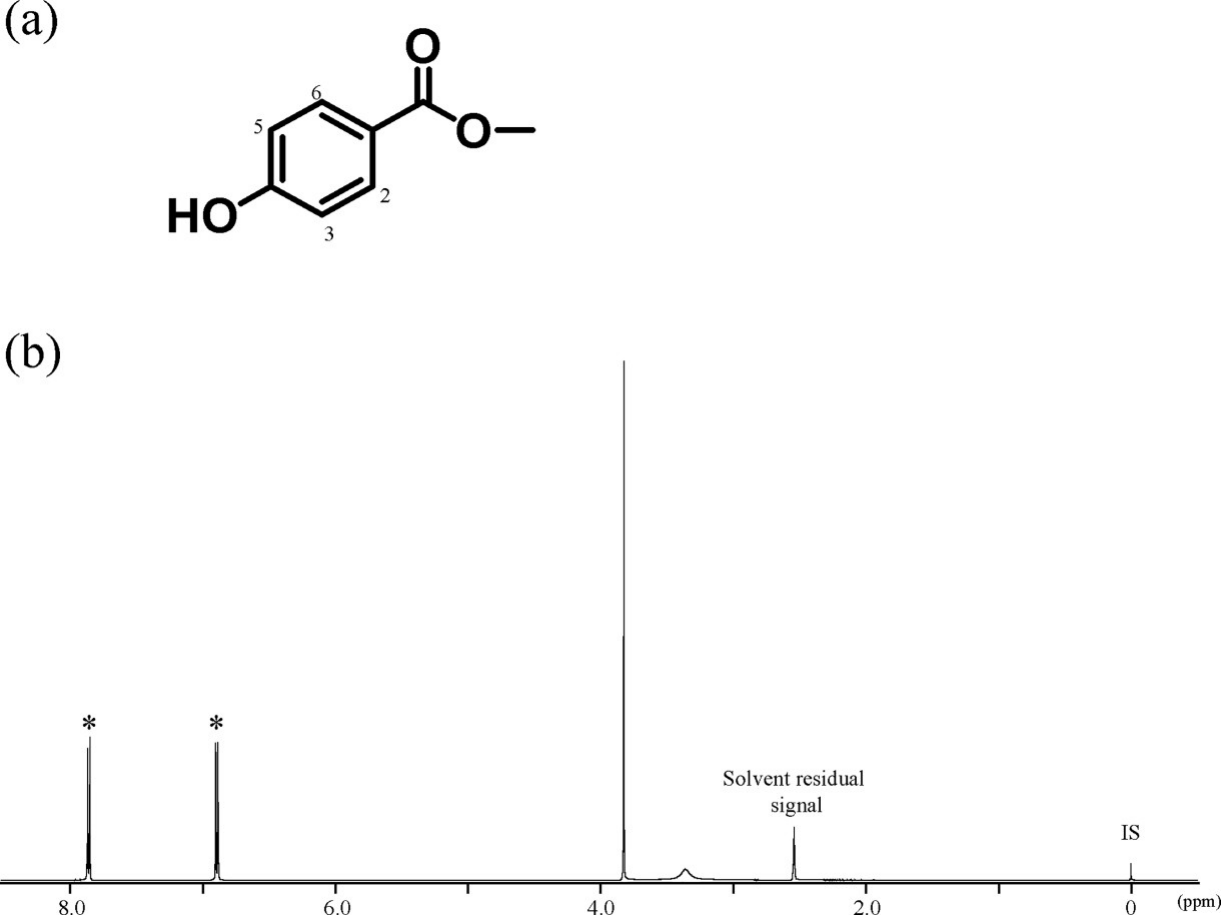
Chemical structure of MHB, and its ^1^H-qNMR spectrum in DMSO-*d*_6_ containing DSS-*d*_6_. IS, internal standard (DSS-*d*_6_).

For MHB, the observed signals were attributed to the methoxy group at δ 3.82 ppm, the two protons from the 3- and 5-positions at δ 6.87 ppm, and the two protons from the 2- and 6-positions at δ 7.84 ppm (Fig 3).

The purity was calculated from the peaks at δ 6.87 ppm and δ 7.84 ppm to be 93.9% and 93.8%, respectively. The purity (93.8%) calculated from the signal at δ 6.87 ppm was adopted as the purity of MHB.

### Calculation of the RMS values of acteoside and pedaliin relative to MHB

To calculate the RMS of acteoside and pedaliin relative to MHB, solutions of acteoside, pedaliin, and MHB were prepared at seven different concentrations ranging from 1.6 to 100 μmol/L based on the purities calculated by ^1^H-qNMR. The HPLC analysis was conducted, and linear regression analysis was made by plotting peak area (y) versus concentration of acteoside, pedaliin, and MHB (x) in μmol/L, respectively.

**Fig 4:**
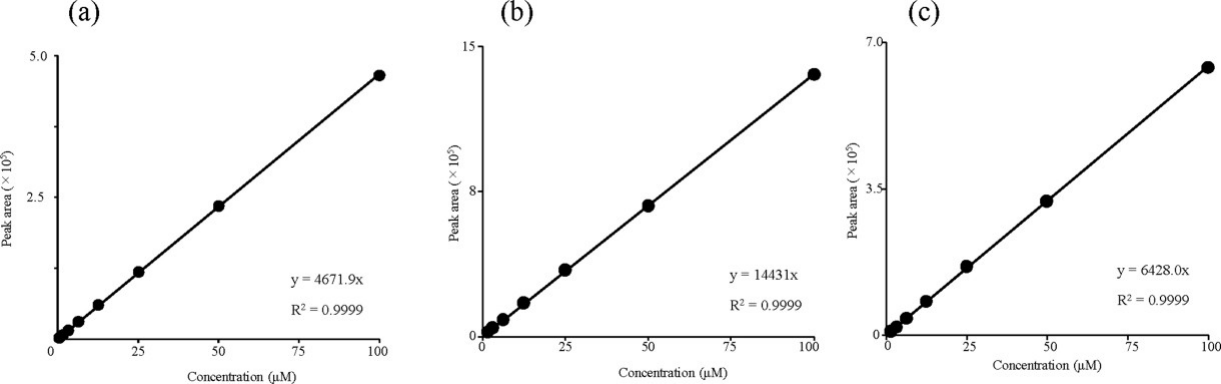
Calibration curves for acteoside (a), pedaliin (b), and MHB (c). The calibration curve slopes were: acteoside, 4671.9; pedaliin, 14431; MHB, 6428. The resulting RMS values relative to MHB were 0.727 (4671.9/6428.0) for acteoside and 2.25 (14431/6428.0) for pedaliin.

Then, the RMS values were calculated from the ratios of the slopes of the respectively obtained calibration curves. Table 1 and Fig 4 confirms that the calibration curves for MHB, acteoside, and pedaliin showed good linearity, with coefficients of determination of 0.9999 and thus, these calibration curves were appropriate for calculating the RMS values.

**Table 1:** Regression data of acteoside, pedaliin, and MHB.

	Calibration equation	Coefficient of determination
Acteoside	y = 4671.9 x	0.9999
Pedaliin	y = 14431 x	0.9999
MHB	y = 6428.0 x	0.9999

### Quantification of acteoside and pedaliin in dried sesame leaf powders and processed foods using the HPLC method and each RMS

**Fig 5:**
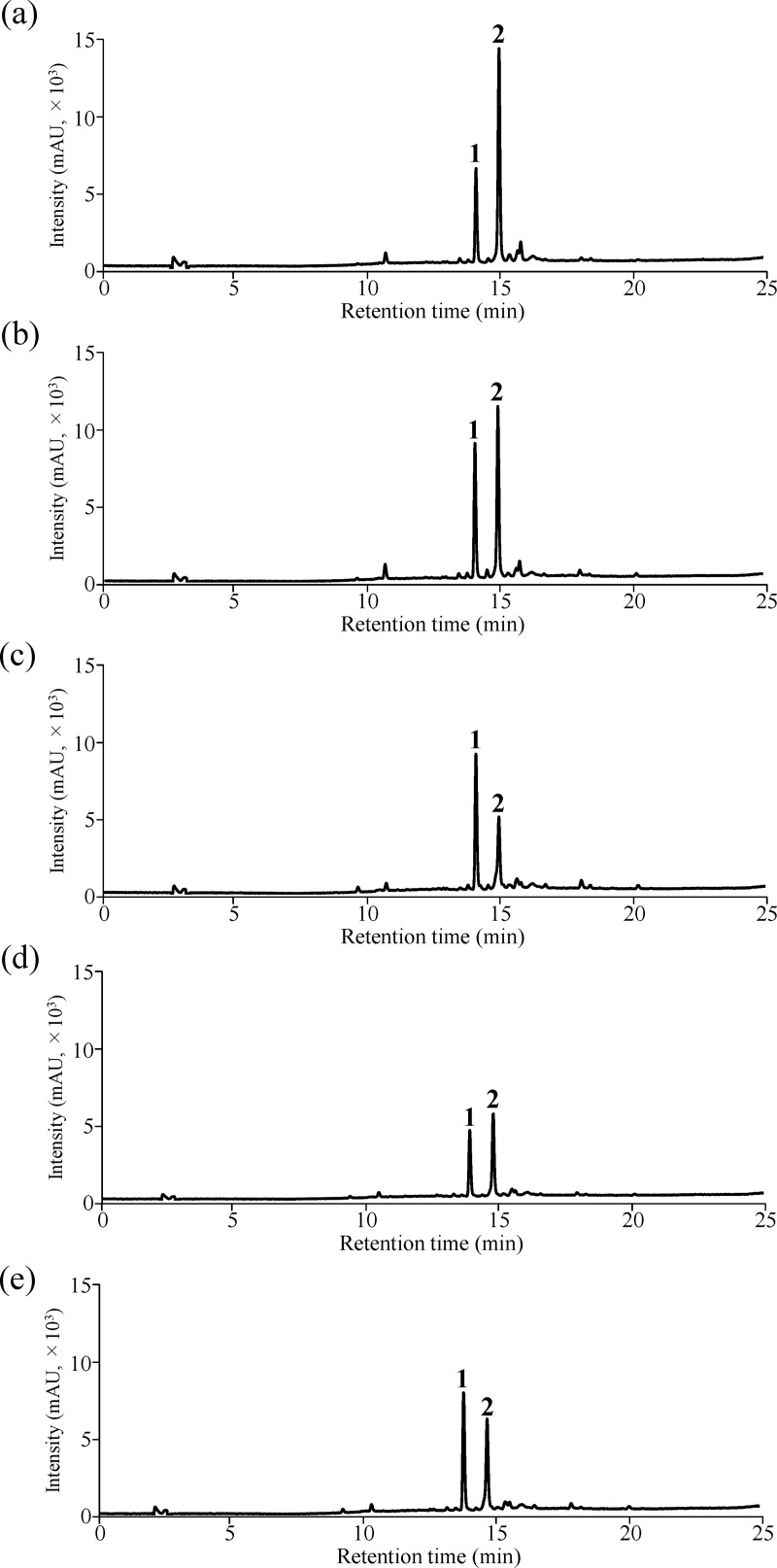
Representative HPLC chromatograms of each samples at 340 nm. Dried sesame leaf powder 1, (b) Dried sesame leaf powder 2, (c) Dried sesame leaf powder 3, (d) Green juice powder 1, (e) Green juice powder 2. The peaks labeled 1 and 2 correspond to acteoside (t_R_: 14.1 min) and pedaliin (t_R_: 15.0 min), respectively.

**Fig 6:**
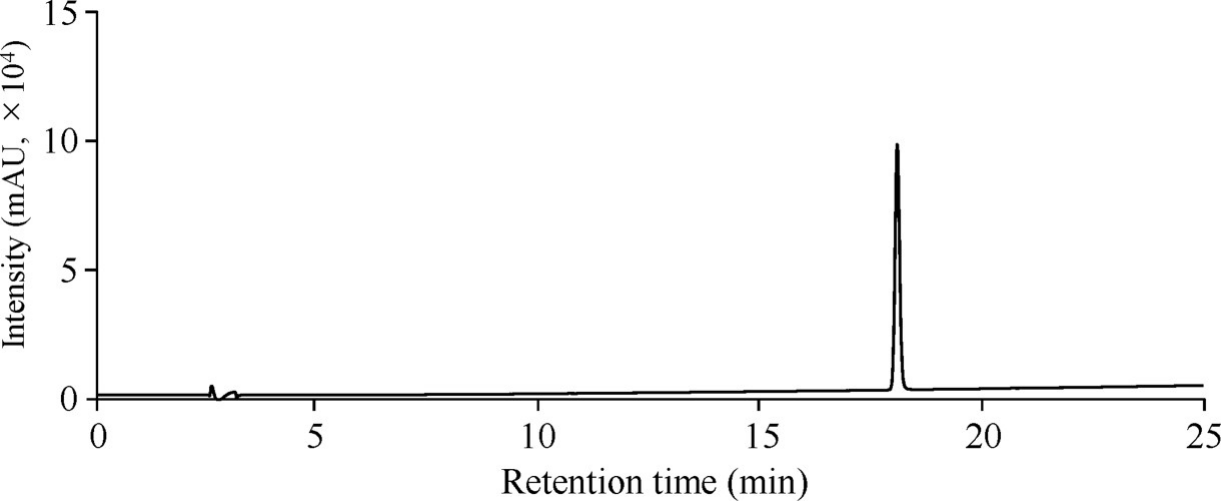
Representative HPLC chromatograms of a MHB (t_R_: 18.2 min) standard solution (100 μmol/L) at 254 nm.

To evaluate the applicability of the calculated RMS for the quantification of acteoside and pedaliin, the HPLC method combined with each RMS was applied to the analysis of three dried sesame leaf powders and two processed foods containing acteoside and pedaliin (n = 3). Furthermore, the quantitative values obtained using the HPLC method and each RMS (RMS method) were compared with the conventional method (an absolute calibration curve method) using acteoside and pedaliin; in this latter case, the purities of acteoside and pedaliin were adjusted based on the ^1^H-qNMR measurements of standards for quantification. Representative chromatograms are shown in Figs 5 and 6.

As shown in Table 2, the mean values of the respective acteoside content calculated using the RMS method were 12.5–125 μg/kg, and that of the pedaliin content were 1.8–10.7 μg/kg. In addition, no significant difference in analyte content was observed for all samples between the RMS and conventional methods by statistical evaluation (*P*<0.01) with the *t*-test (Table 2).

**Table 2:** Comparison of the acteoside and pedaliin contents in samples determined by two methods.

	RMS method				Conventional method			
Sample	Acteoside content (μg/kg)*^1^	RSD (%)	Pedaliin content (μg/kg)*^1^	RSD (%)	Acteoside content (μg/kg)*^1^	RSD (%)	Pedaliin content (μg/kg)*^1^	RSD (%)
Dried sesame leaf powder 1	125±1.9	1.5	9.9±0.03	0.3	126±1.9	1.5	10.0±0.02	0.2
Dried sesame leaf powder 2	76.5±1.0	1.3	10.7±0.4	3.3	77.5±1.0	1.3	10.8±0.4	3.2
Dried sesame leaf powder 3	13.9±0.4	3.2	2.6±0.04	1.5	14.2±0.4	3.1	2.6±0.04	1.5
Green juice powder 1	13.8±0.1	0.7	1.8±0.01	0.5	14.1±0.1	0.7	1.8±0.01	0.5
Green juice powder 2	12.5±0.04	0.3	2.4±0.01	0.2	12.8±0.09	0.7	2.4±0.01	0.2

*^1^ Values represent the mean ± standard deviation of three independent experiments. RSD, relative standard deviation.

Furthermore, precise intra-day data were obtained for all samples using the RMS methods, with the RSDs ranging from 0.2 to 3.3%. Since ^1^H-qNMR was used to determine the purities of quantification standard compounds used in the conventional method, the calculated quantitative values are considered the most accurate. Substantially equivalent quantitative values were obtained using both methods, indicating the accurate quantification of acteoside and pedaliin in dried sesame leaf powders and processed foods using the RMS method.

In acteoside and pedaliin, the commercial reagent or isolate from plant material were used as a reference standard in HPLC and LC-MS/MS analysis for quantification [21–26]. However, the purity or content value assigned from the relative area ratios obtained using GC or HPLC, is not reliable because of differences in the analyte's response factors and the impurities. For accurate quantification, all of these methods require an authentic reference substance, such as a CRM with a proven identity and purity with metrological traceability to the SI. However, there are currently no commercially available CRMs for both analytes. Additionally, no pedaliin reference standard is readily available from commercial sources, negatively impacting conventional relative quantitative methods such as HPLC.

On the other hand, the RMS method allows quantitative analysis for acteoside and pedaliin using each RMS value and an inexpensive and highly stable alternative reference standard such as MHB. Moreover, the respective RMS values presented herein were determined using a reagent whose purity was based on ^1^H-qNMR analysis using certified reference material. Thus, the RMS method was clarified that metrologically accurate quantification of acteoside and pedaliin could be performed without an authentic reference standard of the analyte.

## Conclusions

The RMS method presented in this study is useful for determining the acteoside and pedaliin content of dried sesame leaf powders and processed foods containing these compounds. Using MHB as an alternative reference standard for quantification and the obtained RMS values of 0.727 and 2.25 for acteoside and pedaliin, respectively, quantifies these two compounds accurately. The respective RMS values presented herein were determined using a reagent whose purity was based on ^1^H-qNMR analysis using certified reference material. This ensures SI traceability of the analytical method and allows reliable quantification of acteoside and pedaliin using the provided RMS values without conducting ^1^H-qNMR measurements. This method can accurately measure multiple components using a single alternative reference standard if the analytes' RMS values against the alternative reference standard can be determined, in contrast to conventional methods that require an authentic reference standard for each analyte. As such, the proposed RMS method is a very useful and efficient tool for determining the acteoside and pedaliin content of samples. In addition, because the proposed RMS method is superior from the versatility, accuracy, and analysis cost, the method holds promise for further quantitative analysis applications.
